# Reviewing evidence of the clinical effectiveness of commercially available antivenoms in sub-Saharan Africa identifies the need for a multi-centre, multi-antivenom clinical trial

**DOI:** 10.1371/journal.pntd.0007551

**Published:** 2019-06-24

**Authors:** Julien Potet, James Smith, Lachlan McIver

**Affiliations:** Médecins Sans Frontières Access Campaign, Geneva, Switzerland; Institut de Recherche pour le Développement, BENIN

## Abstract

**Background:**

Snakebite envenoming kills more than more than 20,000 people in Sub-Saharan Africa every year. Poorly regulated markets have been inundated with low-price, low-quality antivenoms. This review aimed to systematically collect and analyse the clinical data on all antivenom products now available in markets of sub-Saharan Africa.

**Methodology/Principal findings:**

Our market analysis identified 12 polyspecific and 4 monospecific antivenom products in African markets. Our search strategy was first based on a systematic search of publication databases, followed by manual searches and discussions with experts. All types of data, including programmatic data, were eligible. All types of publications were eligible, including grey literature. Cohorts of less than 10 patients were excluded. 26 publications met the inclusion criteria. Many publications had to be excluded because clinical outcomes were not clearly linked to a specific product. Our narrative summaries present product-specific clinical data in terms of safety and effectiveness against the different species and envenoming syndromes. Three products (EchiTabPlus, EchiTabG, SAIMR-Echis-monovalent) were found to have been tested in robust clinical studies and found effective against envenoming caused by the West African carpet viper (*Echis ocellatus*). Four products (Inoserp-Panafricain, Fav-Afrique, SAIMR-Polyvalent, Antivipmyn-Africa) were found to have been evaluated only in observational single-arm studies, with varying results. For nine other products, there are either no data in the public domain, or only negative data suggesting a lack of effectiveness.

**Conclusions/Significance:**

Clinical data vary among the different antivenom products currently in African markets. Some products are available commercially although they have been found to lack effectiveness. The World Health Organization should strengthen its capacity to assess antivenom products, support antivenom manufacturers, and assist African countries and international aid organizations in selecting appropriate quality antivenoms.

## Author summary

Snakebite envenomation represents one of the most neglected tropical medical conditions worldwide. Despite high levels of morbidity and mortality associated with snakebite, its neglected nature has compromised the availability and evaluation of antivenom treatment. This review was initiated by Médecins Sans Frontières’ / Doctors Without Borders (MSF) Access Campaign, as the existing antivenom access crisis in sub-Saharan Africa was deepening. This study sought to review the clinical data pertaining to each antivenom product currently available for use in sub-Saharan Africa. 16 different antivenoms were identified. A total of 26 studies met the inclusion criteria. Given the heterogeneity of study methodology and quality, data for individual antivenoms are presented in the form of a narrative analysis. Only two studies reported clinical data collected from randomized controlled trials. Consistent monitoring of the side effects of antivenom treatment was lacking. The absence of good quality data for the majority of antivenoms in sub-Saharan Africa is a major concern. Further robust data collection is required, while urgent investments are needed at the global level to ensure a sustained production of safe and effective antivenom treatment, and its affordable access across sub-Saharan Africa.

## Introduction

More than 100,000 people die from snakebite envenoming every year, associating this neglected tropical medical condition with one of the highest burdens of mortality of all neglected tropical diseases (NTDs). In sub-Saharan Africa alone, snakebites are estimated to cause between 435,000 and 580,000 envenomings, and between 20,000 and 32,000 deaths every year [1]. 30 different species have been found to cause life-threatening envenomings [2]. While six different clinical syndromes are described by the World Health Organization (WHO) [2], field organisations in sub-Saharan Africa, such as Médecins Sans Frontières / Doctors without Borders (MSF), distinguish three major syndromes requiring antivenom therapy: neurotoxic, haematotoxic and cytotoxic (see Table 1). Prompt administration of a safe, effective and geographically appropriate antivenom is the cornerstone of effective snakebite management, although supportive care is crucial too, including assisted ventilation in case of neurotoxic envenoming.

**Table 1:** Main envenoming syndromes requiring antivenom therapy in sub-Saharan Africa.

Syndrome	Symptoms	Medically important snake genera
CYTOTOXIC	Painful progressive swelling, necrosis	Spitting cobras (*Naja spp*.), adders (*Bitis spp*.), saw-scaled/carpet vipers (*Echis spp*.)
HAEMATOTOXIC	aka ‘Viperid syndrome’ Bleeding, non-clotting	Saw-scaled/carpet vipers (*Echis spp*.) Rarely adders (*Bitis spp*.) & Boomslang (*Dispholidus typus*)
NEUROTOXIC	aka ‘Elapid syndrome’ Progressive weakness & paralysis	Non-spitting cobras (*Naja spp*.), mambas (*Dendroaspis spp*.)

Despite the medical need for antivenom treatment, a decades-long antivenom supply crisis continues to affect sub-Saharan Africa [3]. Some locally inappropriate antivenom products, which are not prepared using the venom of snake species found in the sub-region have nevertheless been commercialised in sub-Saharan Africa [4]. In addition, several historical suppliers ceased production of their African antivenom products, citing limited profit. Notably, in 2014 Sanofi-Pasteur produced the last batch of Fav-Afrique, a polyspecific antivenom intended for use in sub-Saharan Africa and marketed for nearly two decades. The uncertain quality and specificity of certain antivenom products in many African countries has eroded the confidence of healthcare workers in antivenom therapy. In order to restore it, better regulation of the antivenom market, and the phase-out of ineffective and locally inappropriate products, is necessary.

The manufacture of snake antivenom products follows a three-step process: immunisation of animals (most often horses) with a mixture of venoms; collection and fractionation of animal plasma, followed by refinement of immunoglobulins. In practice, products differ by the composition of the immunising venom mixtures, the animal immunisation protocols, the fractionation and purification processes, and the fragmentation (or otherwise) and concentration of immunoglobulins. Due to these major differences, the clinical safety and efficacy of an individual product cannot be extrapolated to another product.

While the WHO recommends the evaluation of snake antivenom products in pre-clinical and clinical studies before their commercialisation [5], the number of robustly-designed clinical trials on snakebite and antivenom therapy is extremely limited [6]. In sub-Saharan Africa, half of the clinical studies listed in a recent review were observational single-arm studies, which preclude any direct comparison between antivenom products [7]. Acknowledging this limitation, we nonetheless proceeded to review the available clinical data related to each individual antivenom product currently available for use in sub-Saharan Africa. The product-by-product clinical data summaries in this review do not allow for head-to-head comparisons between products. However, they may be helpful in determining the risk-benefit of each individual product in a specific region, dependent on the local distribution of snake species.

## Methods

### List of commercially available antivenom products

In 2014 we established a list of antivenom products commercially available in sub-Saharan Africa. The list was regularly updated and is presented in (Table 2). A number of different sources were used: the WHO database of venomous snakes, direct enquiries to suppliers, discussions with experts, and reporting from MSF teams at the project level. In comparison to a list published by Brown during a similar study [8], a number of major changes are evident, reflecting the fluctuant nature of the African antivenom market: the production of several products has ceased, while new products have reached markets in sub-Saharan Africa. Although the manufacture of Fav-Afrique was ceased by Sanofi-Pasteur, we included the product in our list because another company, MicroPharm, has announced its intention to re-launch the product [9]. In order to improve readability, an abridged name was attributed to every individual product and used hereafter in the manuscript.

**Table 2:** Commercially available sub-Saharan African antivenom products, 2014–2018.

Brand name *(Abridged name in manuscript)*	Company	Country of production	Species venoms neutralized according to product insert	Type of IgG
**Polyspecific**				
ASNA antivenom C *(ASNA-C)*	Bharat Serums and Vaccines	India	*Bitis arietans*, *B*. *gabonica*, *B*. *nasicornis*, *Dendroaspis angusticeps*, *D*. *jamesoni*, *D*. *polylepis*, *Echis carinatus*, *Naja haje*, *N*. *melanoleuca*, *N*. *nigricollis*, *N*. *nivea*	F(ab)'2—equine
ASNA antivenom D *(ASNA-D)*	Bharat Serums and Vaccines	India	*Bitis arietans*, *B*. *gabonica*, *B*. *nasicornis*, *Dendroaspis angusticeps*, *D*. *jamesoni*, *D*. *polylepis*, *Echis ocellatus*, *Naja haje*, *N*. *melanoleuca*, *N*. *nigricollis*, *N*. *nivea*	F(ab)'2—equine
EchiTabPlus *(ET-Plus)*	ICP	Costa Rica	*Bitis arietans*, *Echis ocellatus*, *Naja nigricollis*	Intact IgG—equine
Inoserp Pan-Africa *(Inoserp-P)*	Inosan	Mexico/Spain	*Bitis arietans*, *B*. *gabonica*, *B*. *rhinoceros*, *Dendroaspis angusticeps*, *D*. *jamesoni*, *D*. *polylepis*, *D*. *viridis*, *Echis leucogaster*, *E*. *ocellatus*, *E*. *pyramidum*, *Naja haje*, *N*. *katiensis*, *N*. *melanoleuca*, *N*. *nigricollis*, *N*. *nivea*, *N*. *pallida*	F(ab)'2—equine
Antivipmyn-Africa *(Antivip-A)*	Instituto Bioclon / Silanes	Mexico	*Bitis arietans*, *B*. *gabonica*, *B*. *rhinoceros*, *Dendroaspis angusticeps*, *D*. *jamesoni*, *D*. *polylepis*, *D*. *viridis*, *Echis leucogaster*, *E*. *ocellatus*, *E*. *pyramidum*, *Naja haje*, *N*. *katiensis*, *N*. *melanoleuca*, *N*. *nigricollis*, *N*. *nivea*	F(ab)'2—equine
Snake Venom Antiserum (PanAfrica) *(Premium-A)*	Premium Serums	India	*Bitis arietans*, *B*. *gabonica*, *B*. *nasicornis*, *B*. *rhinoceros*, *Dendroaspis angusticeps*, *D*. *jamesoni*, *D*. *polylepis*, *D*. *viridis*, *Echis carinatus*, *E*. *leucogaster*, *E*. *ocellatus*, *Naja nigricollis*, *N*. *haje*, *N*. *melanoleuca*	F(ab)'2—equine
Snake Venom Antiserum (Central Africa) *(Premium-CA)*	Premium Serums	India	*Bitis rhinoceros*, *Echis carinatus*, *Daboia russelli*, *Dendroaspis polylepis*	F(ab)'2—equine
Fav-Afrique *(FAV-A)*	Sanofi-Pasteur	France	*Bitis arietans*, *B*. *gabonica*,*Dendroaspis jamesoni*, *D*. *polylepis*, *D*. *viridis*, *Echis leucogaster*, *E*. *ocellatus*, *Naja haje*, *N*. *melanoleuca*, *N*. *nigricollis*	F(ab)'2—equine
SAIMR Polyvalent *(SAIMR-Poly)*	SAVP	South Africa	*Bitis arietans*, *B*. *gabonica*, *Dendroaspis angusticeps*, *D*. *jamesoni*, *D*. *polylepis*, *Hemachatus haemachatus*, *Naja annulifera*, *N*. *melanoleuca*, *N*.*mossambica*, *N*. *nivea*	F(ab)'2—equine
Snake Venom Antiserum Polyvalent (equine) *(VACSERA-Poly)*	VACSERA	Egypt	*Bitis arietans*, *B*. *gabonica*, *Cerastes cerastes*, *C*. *vipera*, *Echis carinatus*, *E*. *coloratus*, *Macrovipera lebetina*, *M*. *palestinae*, *Naja haje*, *N*. *melanoleuca*, *N*. *mossambica*, *N*. *nigricollis*, *N*. *oxiana*, *Pseudocerastes persicus*, *Vipera ammodytes*, *Vipera xanthina*, *Walterinnesia aegyptia*	F(ab)'2—equine
Afriven 10, Snake Venom Antiserum (African) *(VINS-A)*	VINS Bioproducts	India	*Bitis arietans*, *B*. *gabonica*, *Dendroaspis jamesoni*, *D*. *polylepis*, *D*. *viridis*, *Echis leucogaster*, *E*. *ocellatus*, *Naja haje*, *N*. *melanoleuca*, *N*. *nigricollis*	F(ab)'2—equine
Anti Snake Venom Serum Central Africa *(VINS-CA)*	VINS Bioproducts	India	*Bitis gabonica*, *Echis carinatus*, *Daboia russelli*, *Dendroaspis polylepis*	F(ab)'2—equine
**Monospecific**				
EchiTabG *(ET-G)*	MicroPharm	UK	*Echis ocellatus*	IgG—ovine
SAIMR Echis ocellatus / Echis pyramidum *(SAIMR-Echis)*	SAVP	South Africa	*Echis carinatus*, *E*. *ocellatus*, *E*. *coloratus*, *Cerastes spp*.	F(ab)'2—equine
SAIMR-Boomslang *(SAIMR-Boom)*	SAVP	South Africa	*Dispholidus typus*	F(ab)'2—equine
Snake venom antiserum Echis ocellatus *(VINS-Echis)*	VINS Bioproducts	India	*Echis ocellatus*	F(ab)'2—equine

For each individual product, Table 2 lists the different species that are mentioned on the product insert. This must be viewed with much caution. According to good practice, the list of species venoms used in the immunising mixture (and therefore *specifically* neutralised by the antivenom) should be clearly indicated on the product insert. This should be distinguished from the list of species venoms that can be neutralised by *paraspecific* activity. However, some product inserts simply provided a list of species venoms, without necessarily clarifying whether they had been used in the immunising mixture.

Likewise, some manufacturers have yet to adopt the most recent taxonomic changes, for example the identification of new species within the *Echis* genus. Some of the antivenoms commercialized in sub-Saharan Africa are actually raised against venom of *Echis carinatus*, a species endemic in Asia, but not in Africa. While all carpet vipers of Africa and South Asia used to be classified as *Echis carinatus*, distinctive *Echis* species have now been identified (i.e. *Echis ocellatus*, *E*. *pyramidum*, *E*. *leucogaster*) and the composition of the immunizing mixtures of the different antivenoms intended for us in sub-Saharan Africa should take into consideration those changes.

Finally, only a small minority of antivenom manufacturers report the geographical origins of the venoms used in the immunizing mixture, a good practice that should be generalized as there may be major intra-species venom variations between specimens of a given species coming from different geographical locations.

The antivenom products that are included in this review have very different profiles. Most of them contain F(ab)’2 fragments of equine immunoglobulins, but one product contains Fab fragments of ovine immunoglobulins, and another contains intact equine immunoglobulins. Most importantly, very different venom mixtures are used for the preparation of the different products. A few products are monospecific antivenom products, which are raised against the venom of only one snake species. Many products are polyspecific “pan-African” products that are raised against the venoms of the medically most important snakes across different sub-regions of sub-Saharan Africa. Between these two models, some antivenoms have a narrower polyspecificity; they are raised against a limited number of venoms of medically important snakes of a specific sub-region of sub-Saharan Africa.

### Publication search strategy

The first phase of our search strategy was a database search. We searched for publications related to any of the above antivenom products on PubMed (Medline), Cochrane, Embase, Web of Science, Scopus, as well as on regional databases (AJOL, Scielo). The keyword “antiven*” was used in association with: a) geographical keywords (“Africa” or regional country names); b) the names of the companies and products listed in (Table 2); or c) the taxonomic names of the medically most important African snake species. The search was performed on July 24^th^ 2015.

The second phase of our search strategy employed additional search methods: the references of some specific articles and reviews were manually reviewed; experts were asked to review their own personal archives for additional studies; and conference abstract books were searched manually, including the most recent conference abstract books of the International Society of Toxinology and all conference abstract books of the African Society of Venimology. A complementary search was performed in January 2016 on PubMed alone with the keywords “VACSERA” and “Premium” as these manufacturers had not been included in the list of suppliers at the time of the initial database search. Finally, on February 5^th^ 2018, an additional PubMed search with the unique keyword “antiven*” was performed for the period since July 24^th^ 2015, in order to capture any additional papers featured in peer-reviewed journals since the first database search.

All types of clinical data were eligible for inclusion: randomized controlled trials, case-control studies, observational cohort studies, case series, and programmatic data. All patient populations of all ages were included. Studies reporting less than 10 patients per antivenom product were excluded. No date restrictions were applied. All forms of publication were eligible for inclusion: peer-reviewed journal articles, university theses, conference abstracts, and posters. Only publications in French and English were included.

In the event of duplicate publications, defined as different publications related to the same group of patients, the chosen study was either the most recent, or more often, the study with the largest published dataset.

### Categorisation of studies and assessment of quality of evidence

The standardised Newcastle-Ottawa Scale was initially proposed to evaluate the quality of the included studies. However, following capture of the relevant papers it was deemed not worthwhile, as the scale was not well adapted to the overall very low quality of selected studies. Instead we classified studies according to four categories that were adapted from Chippaux’s categorisation of clinical studies on snakebite envenomings [7]:

1. Anecdotal clinical report (ACR): exclusively retrospective study; or prospective study with very limited data on antivenom clinical effectiveness and safety (i.e. very small cohort, did not specifically aim to assess the therapeutic effect or safety of antivenom);
2. Observational cohort study (OCS): prospective study that methodically analyses the therapeutic effects and/or the safety of an antivenom;
3. Non-randomised comparative clinical study (CCS): prospective study that methodically analyses the therapeutic effects and/or the safety of several antivenoms; patient allocations were not-random, but instead depended on other circumstances (i.e. shortages, symptomatology);
4. Randomised clinical trial (RCT): prospective study that methodically analyzes the therapeutic effects and/or the safety of several antivenoms; patient allocations were random.

While randomised clinical trials provided clinical evidence of the highest quality, anecdotal clinical studies provided the evidence of the lowest quality. Observational cohort studies and non-randomised comparative clinical studies provided evidence of moderate quality. However, heterogeneity was noted within each grouping. A wealth of clinical data could be extracted from some retrospective reports, while some prospective cohort studies were reported so poorly that only minimal data could be extracted.

## Results

**Fig 1:**
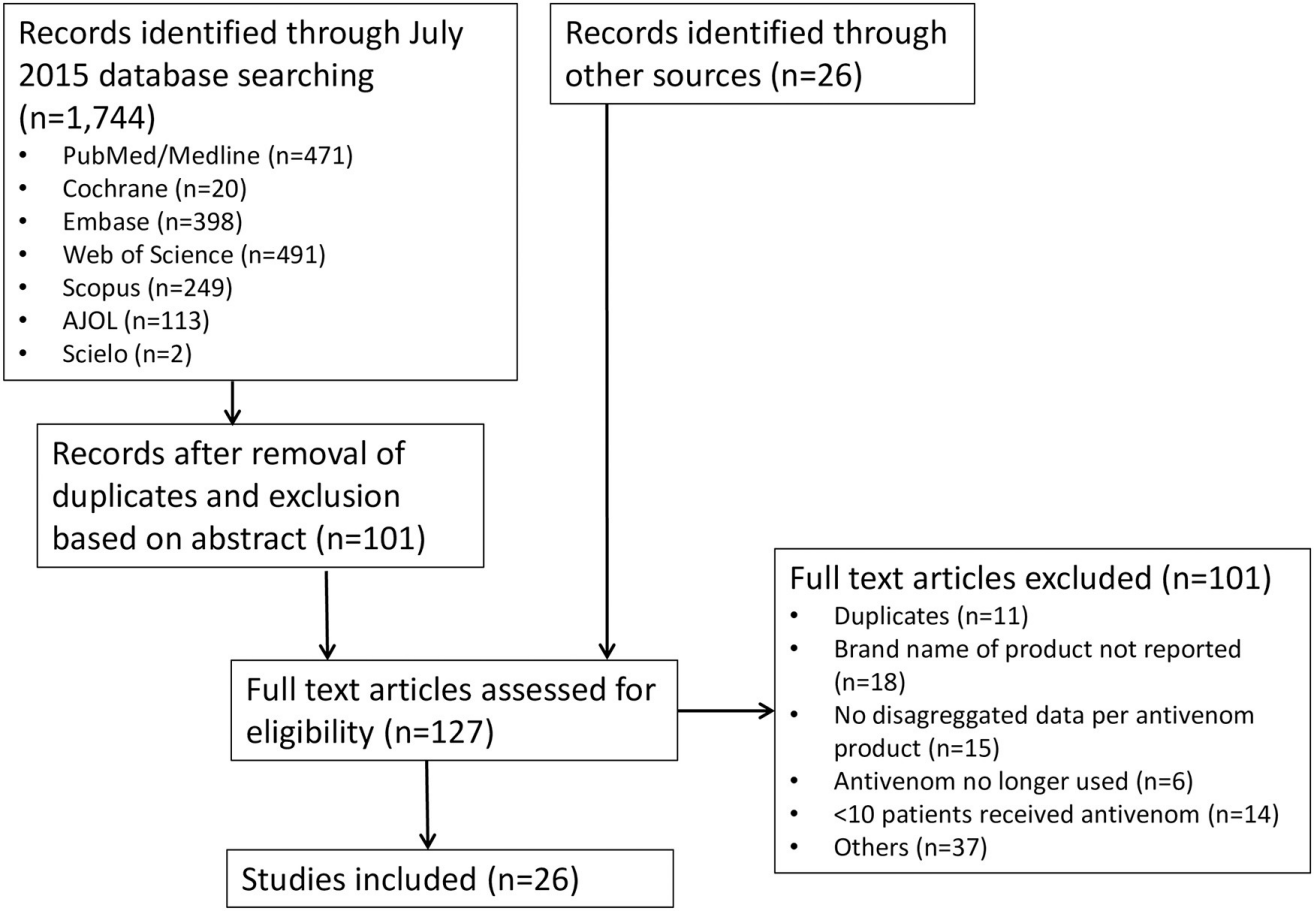
Search strategy.

During the first phase of the search strategy a total of 1744 articles were found. The vast majority of publications at this stage did not meet our two major inclusion criteria: namely, studies examining the clinical efficacy and/or effectiveness of antivenoms, and involving antivenoms currently marketed for use in sub-Saharan Africa. Following abstract screening, 101 publications were reviewed in full. During the second phase of the search strategy 26 additional publications were identified. In total, the full text of 127 publications were considered against the key inclusion and exclusion criteria, of which 26 full-text publications were included in the final review. The main reasons for exclusion are detailed in Fig 1.

Table 3 lists the main characteristics and data extracted from the 26 included studies. Based on the extracted data, the following product-by-product narrative summaries were prepared.

**Table 3:** Data extracted from included studies.

Country Year [REF]	Antivenom products	Study design	Snake species, syndromes	Antivenom Cohort Size	Quantity (mean # of vials, mean mL, # of repeated doses)	CFR (among treated)	Hematotoxicity outcomes	Neurotoxicity outcomes	Adverse side events
Nigeria 2010 [10]	ET-Plus ET-G	RCT	Echis ocellatus, haematotoxic	400	33/194 (17.0%): >1 dose of 3 vials of ET-Plus 36/206 (17.5%): >1 dose of 1 vial of ET-G	No fatalities (9 patients died after supply rupture in 2009)	Blood coagulability restored at 6 hours in 161/194 (83%) of ET-Plus, 156/206 (75.7%) of ET-G (p = 0.05). Recurrent incoagulopathy in 24 patients (7 ET-Plus, 17 ET-G) (p = 0.04)	Not reported	ET-Plus 50/194 (25.8%), ET-G 39/206 (18.9%) (p = 0.06). ET-Plus 21 (10.8%) vs ET-G 11 (5.3%) severe GI or bronchospasm (p = 0.03) No pyrogenic. 5 late reactions (ET-Plus), 3 (ET-G)
CAR 2017 [11]	FAV-A ET-Plus SAIMR-Poly	CCS	311 hematotoxic (mainly caused by Echis ocellatus), 12 neurotoxic, 16 cytotoxic, 8 unclassified, 375 non-envenomed	337 out of 722 27 (FAV-A) 306 (ET-Plus) 4 (SAIMR-Poly)	78% received >1 initial dose of 2 vials of FAV-A 27% received >1 initial dose of 4 vials of ET-Plus	FAV-A: 2/27 (7%) deaths, both deaths in neurotoxic cases ET-Plus: 1/306 (0.3%), death in cytotoxic case	All patients treated for hematotoxic syndrome survived	FAV-A: 2/2 treated for neurotoxic syndrome died ET-Plus: not used in neurotoxic cases	ET-Plus: immediate hypersensitivity reaction in 21 of 306 (6.9%); infusion was stopped in 13 (4.2%)
Nigeria 2011 [12]	ET-Plus ET-G	ACR	Predominantly Echis ocellatus	6,687 total; unclear how many received treatments	Not reported	94 deaths among 6,687 cases, but unclear which treatment (if any) they received	Not reported	New CNS features predictive of mortality	Not reported
Cameroon 1999 [13]	FAV-A	OCS	Substantial number of Echis ocellatus. Predominantly haematotoxic	46 of 61	37ml +/- 4	No fatalities	Not reported	Not reported	Two patients (4.3%) showed minor immediate adverse related to FAV-A; no other treatment-related adverse event occurred. No patient had serum sickness.
Ghana 2008 [14]	FAV-A ASNA-C	CCS	Predominantly Echis ocellatus, haematotoxic	344 (278 FAV-A, 66 ASNA-C)	22% FAV-A required repeat doses, 56% ASNA-C. 5.2v (FAV-A) vs 11.7v (ASNA-C)	Mortality rate 1.8% FAV-A vs 12.1% ASNA-C	1 or 2 doses achieved normalisation in 79% of patients following FAV-A vs 22% with ASNA-C.	Not reported	Allergic reaction on AV administration in 5 patients, all following ASNA-C. All signs of anaphylactic shock, one also skin reaction. Two deaths. All patients received routine prophylaxis.
Chad 2006 [15]	FAV-A (IPSER-A) (SII-CentralAfrica)	CCS	Assumed Echis ocellatus predominantly, haematotoxic, cytotoxic	288 total 60 (FAV-A)	1.9v +/- 0.24 (FAV-A)	6.7% (4/60) (Fav-A)	Mean "clotting recovery time": 9.3d (FAV-A)	Not reported	Not reported
CAR 2015 [16]	FAV-A Antivip-A	CCS	Many cases caused by Echis ocellatus	644 (Fav-A) 50 (Antivip-A)	82% only one dose of 1 or 2 vials (FAV-A) 38% received only one dose of 2 vials (Antivip-A)	FAV-A: 3/644 (0.5%) deaths Antivip-A: 5/50 (10%) deaths. 2d dose not administered timely in 4/5 deaths	FAV-A: not reported Antivip-A: 10/13 were still bleeding within two hours after first dose of two vials	Not reported	Not reported in FAV-A group 2/50 (4%) in Antivip-A group
Djibouti 2008 [17]	FAV-A	OCS	Echis pyramidum All 31 patients with incoagulable blood; 9/31 with external bleeding	31	30ml +/- 10ml	0/31 (0%)	Mean time to restore coagulation: 8h +/- 4h 1/31 with limb necrosis 3/31 had finger amputated 1/31 received dialysis	Not reported	No adverse events
Djibouti 2007 [18]	FAV-A (BEN-Pasteur)	ACR	Echis pyramidum All patients with incoagulable blood	62 (33 IPSER-A; 29 FAV-A)	2.5v +/-1.5 18/29 patients received >1 initial dose of 1 or 2 vials of FAV-A	No fatalities	Among 25 with incoagulable blood treated w/ FAV-A, coagulation was restored w/ 1 vial in 4 (16%), w/ 2 vials in 9 (36%), w/ >2 vials in 12 (48%)	Not reported	1/29 treatd with FAV-A had allergic reaction
Djibouti 2018 [19]	FAV-A	ACR	Hematotoxic (most likely Echis pyramidum, possibly Bitis arietans & Dispholidus typus)	14	2 vials in 3 patients, 3 or 4 vials in 11 patients	No fatalities	All 14 patients with coagulopathy at admission, including 5 patients with bleeding. Coagulopathy monitored by TEG & conventional coagulation assays. Hemostasis parameters remained very disturbed during the first 72 h.	Not reported	Not reported
Nigeria 2012 [20]	ET-G ASNA-C	ACR	Echis ocellatus, haematotoxic	5367 (2669 treated 2009, 2698 in 2010)	Not reported	82/5,367 deaths (1.52%), unclear which (if any) antivenom was received Survival in group who received ET-Plus is 99.0%, but no absolute number is mentioned	Nil coagulability with ASNA-C	Not reported	ASNA-C: all suffered anaphylaxis (>200 patients)
Benin 2007 [21]	Antivip-A	OCS	Predominantly haematotoxic / cytotoxic	289	3.8v +/- 2.6	9 deaths (3.1%) - 6 with serious complications at admission, 1 following shortage of AV, 2 with inexplicable death despite early arrival	Bleeding in 138 patients. Bleeding arrested in 60% of patients within 2 hours, 80% within 24 hours.	Not reported	Unexpected events in 39 (13%) of patients. No serum sickness in 77 patients evaluated at 3 weeks.
Guinea 2012 [22]	Antivip-A	OCS	83% cytotoxic/mild haematotoxic 17% Neurotoxic	150 of 228 total	1.4v +/- 1.0 In neurotoxic subgroup: 2.6v +/- 0.6	4 deaths, 2.7% (all in neurotoxic subgroup)	No case of systemic haemorrage in this cohort. Most cases classified as viperid envenomings caused no bleeding or only minor local bleeding	4 deaths out of 26 cases (2 w/ respiratory paralysis at admission) No capacity for ressuscitation	10/150 (6.7%): prurit and/or rash, urticaria, diarrhea, cough 5 within 1h after administration 5 several days after administration
Giunea 2013 [23]	Antivip-A	OCS	African elapidae, neurotoxic	Total: 77 44 prospective / 33 retrospective	Low initial dose group—26.2ml +/- 5.9, high initial dose group—41.2ml +/- 2.3	27.3% (untreated), 15.4% (low initial dose), 17.6% (high initial dose)	Not reported	See CFR	Not reported
Benin, Guinea 2015 [24]	Inoserp-P	OCS	Benin: >90% haematotoxic Guinea: 88% cytotoxic/mild haematotoxic; 12% neurotoxic	Benin: 100 of 100 Guinea: 109 of 165	Benin: 1.7v Guinea: 1.1v (2.3v in neurotoxic subgroup)	Benin: 4/100 (4%); 1 immediately after admission, 1 with Hb = 3g at admission; 3 out of 4 received sub-optimal dose (<2vials) Guinea: 1/109 (1%)	Benin: Blood coagulation restored in 98% of patients within 24h	Guinea: CFR: 1/13 (8%) in neurotoxic subgroup	Benin/Guinea: 17 /209 (8%) with prurit and/or urticaria, nausea, cough, dizziness, dyspnea; all resolved w/ antihistaminics or corticoids; likely underreporting of adverse events in one site
South Africa 2004 [26]	SAIMR-Poly	ACR	Cytotoxic predominantly	12 of 333 total	5/7 with progressive weakness received doses 60-200ml, 5/281 with progressive swelling received 18-50ml	No fatalities	Not reported	Not reported	Adverse reactions in 5/12 (described only for 4)
South Africa 1998 [27]	SAIMR-Poly	OCS	Haematotoxic—swelling / coagulopathy	17 of 147	Reported by patient	No fatalities	Not reported	Not reported	13 patients severe early anaphylactoid reactions (generalised urticaria—12, angio-oedema—3, bronchospasm—2, hypotension—2). 3 other possible anti-venom related responses.
South Africa 2009 [28]	SAIMR-Poly	OCS	Puff adder likely most common. Haematotoxic cases.	22 of 243	Initial 4 vials (40ml). Further doses given, but unclear how much.	No fatalities	Not reported	Not reported	Only 7 patients did not have side effects. Allergic response in 4, anaphylaxis in 6. One referral to tertiary care following anaphylaxis.
Tanzania 2010 [29]	SAIMR-Poly	OCS	Puff adder most common, followed by red spitting cobra. Cytotoxic	42 of 85	40 cases: one 10ml vial only. One severe neurotoxic required 8x 10ml. Other neurotoxic required 2x 10ml.	No fatalities among treated group	Not reported	Not reported	Adverse reactions in 7 (8%): urticarial rash, nausea, tachycardia and / or tachypnoea.
South Africa 1987 [30]	SAIMR-Poly	ACR	In treated group: Mild-to-moderate cytotoxic: 2 Severely cytotoxic: 8 Neurotoxic: 30 Eye envenomation: 1	41 of 251	Not reported	In treated group: Mild-to-moderate cytotoxic: 0/2 Severely cytotoxic: 0/8 Neurotoxic: 2/30 Eye envenomation: 0/1	Not reported	Not reported	1 anaphylaxis reported among 41 treated
South Africa 1994 [31]	SAIMR-Poly	ACR	In the 20 patients with systemic envenomings: 6 neurotoxic, 3 hematotoxic, 11 non-specific/mixed	10 of 81	Not reported	3 of 10 patients treated w/ antivenom died. All were children <10y.	Not reported	Not reported	Adverse reactions in 4/10, requiring ressuscitation and parenteral adrenalin
Nigeria 1976 [32]	SAIMR-Echis (Behring-Poly)	OCS	Echis ocellatus, haematotoxic	16 of 23 were treated exclusively w/ SAIMR-Echis	2.6v	No fatalities (0/16)	Bleeding stopped for the majority within 24 hours. Recurrent bleeding at between Day3-6 in 4/16.	Not reported	Not reported
Nigeria 1977 [33]	SAIMR-Echis (Behring-Poly) (Pasteur-Echis)	CCS	Echis ocellatus, haematotoxic	107 of 115 including 38 only SAIMR-Echis 10 SAIMR-Echis & other	18.5ml (SAIMR-Echis subgroup)	0/48 in SAIMR-Echis subgroup	Not reported in a disagreggated manner by antivenom	Not reported	14/48 (29%) who received SAIMR-Echis had reactions
Nigeria 1974 [34]	SAIMR-Echis (Behring-Poly)	RCT	Echis ocellatus, haematotoxic	46 (23 SAIMR-Echis, 23 Behring-Poly)	15.2ml (SAIMR-Echis subgroup)	No fatalities	Average time from treatment to permanent restoration of coagulability significantly better in SAIMR-Echis	Not reported	4/23 who SAIMR-Echis had Immediate hypersensitivity
Ethiopia 2016 [35]	VACSERA-Poly	ACR	23 hematotoxic (w/ prolonged clotting time), 4 w/ normal clotting time	23 of 27	Not reported with accuracy; in most cases no more than 1–3 vials, because of short supply	4/23 deaths (17%)	Not reported	Not reported	Not reported
Senegal 2019 [25]	Inoserp-P	OCS	34 mild envenoming, 24 haematotoxic, 8 neurotoxic	63 of 66	92 vials for 63 patients (1.5v per patient)	2/63 deaths (3%)	Bleeding was stopped and blood-clotting was restored within 24 hours in 87.5% of patients	Reversal of neurotoxicity symptoms in 75% of patients	1 anaphylaxis ‘possibly’ related and 1 dizzyness ‘probably’ related to antivenom

Abbreviations: RCT, randomized clinical trial; CCS, non-randomized comparative clinical study; OCS, observational cohort study; ACR, anecdotal clinical report

### ET-Plus

ET-Plus was trialed in a large-scale RCT against ET-G in northern Nigeria to treat carpet viper envenomings (*Echis ocellatus*) [10]. 194 victims received ET-Plus. Results were good: blood coagulability, which is typically altered in carpet viper envenomings, was restored in 83% of patients within 6 hours; no fatalities were recorded. Of concern, adverse events were recorded in more than one-fourth of patients, including severe adverse events in one-tenth. An initial dose of three vials of ET-Plus was found to be a little more effective than an initial dose of one vial of ET-G, and a little less safe.

A prospective study in Paoua, Central African Republic [11], where *Echis ocellatus* is believed to be the medically most important species, was suggestive of ET-Plus effectiveness: there was only one death among the 306 victims of cytotoxic or hematotoxic envenomings who received ET-Plus. An immediate hypersensitivity reaction was seen in 21 patients (6.9%).

A retrospective survey in Nigeria found that case fatality due to snakebite after introduction of ET-Plus was low [12], but these results should be interpreted with caution given the low quality of programmatic data.

*Summary*: ET-Plus was found to be satisfactorily clinically effective against *Echis ocellatus* envenomings; there is no clinical evidence on its effectiveness for envenomings caused by other species; the rate of adverse events appears moderate.

### ET-G

ET-G was trialed in a large-scale RCT against ET-Plus in northern Nigeria to treat carpet viper evenomings (*Echis ocellatus*) [10]. 206 victims received ET-G. Efficacy was found to be good with an initial dose of one vial, although not as good as with an initial dose of three vials of ET-Plus: blood coagulability was restored in 76% of patients within 6 hours; no fatalities were recorded; adverse events were recorded in less than 20% of patients, of which severe adverse events were recorded in fewer than 5% of patients. Further to the above, an initial dose of one vial of ET-G was found to be a little less effective than an initial dose of three vials of ET-Plus, but a little safer. ThusET-G would seem to be more dose-effective and safe than ET-Plus to treat envenoming by *Echis ocellatus*.

A low-quality retrospective survey was conducted in Nigeria following the introduction of ET-G [12], which seems to indicate that mortality due to snakebite was low in patients treated with this antivenom. These results should be interpreted with much caution.

Summary: ET-G was found to be very clinically effective against *Echis ocellatus* envenomings, the only species against which it is indicated, as clotting was restored in the majority of patients treated with just one vial. The rate of adverse events seems low-moderate.

### FAV-A

FAV-A was tested in four good quality prospective cohort studies in West Africa. In northern Cameroon [13], in a region where *Echis ocellatus* envenomings are common, FAV-A was used successfully in all 41 patients, and only two minor adverse events were attributed to the antivenom. In a similar setting with 278 patients in Central Ghana [14], FAV-A was associated with a low mortality rate of 1.8%; only 22% of patients required repeat antivenom doses. In southern Chad [15], 4 of 60 patients treated by FAV-A died; of note, no repeat antivenom doses could be given to patients who would need them, due to limited resources. In a prospective study in Paoua in CAR [11], a region where envenomings are caused predominantly by *Echis ocellatus* and occasionally by other species including neurotoxic elapids, there were two deaths among the 27 patients treated with FAV-A, both with features of neurotoxic syndrome. This raises questions of the effectiveness of FAV-A against elapid neurotoxic envenomings. In addition, 78% of snakebite victims in this cohort had to receive repeat doses of FAV-A. This contrasts with a previous retrospective analysis of 644 patients in Paoua [16], which found that FAV-A was associated with a low mortality rate of 0.5%.

In three cohorts in Djibouti [17, 18, 19], FAV-A was found to restore blood coagulability following bites by *Echis pyramidum*. There were no fatalities among the total of 74 patients treated.

In all of these studies, few adverse events were reported. But this apparent good safety profile should be interpreted with caution, as the quality of monitoring of adverse reactions varied across the studies.

Summary: FAV-A was found to be clinically effective against African carpet viper envenomings, notably *Echis ocellatus* and *Echis pyramidum*. There are few clinical indications related to its effect on envenomings caused by other species. The rate of adverse events seems low.

### ASNA-C

In a good quality post-marketing surveillance study in central Ghana [14], a region where envenomings are often caused by *Echis ocellatus*, ASNA-C was associated with a very high mortality rate of 22%, with a mean number of 11.7 vials used per patient. More than half of the 66 treated patients had to be administered repeat antivenom doses. Five cases of anaphylactic shock were reported amongst 66 patients.

In a programmatic setting in Nigeria, from a low quality report [20], ASNA-C was believed to be ineffective in restoring blood coagulopathy, and to cause many cases of allergic reactions.

Summary: ASNA-C was found ineffective at neutralising envenomings caused by *Echis ocellatus*. The rate of severe adverse events appears high.

### Antivip-A

Two observational cohort studies were conducted in a setting where snakebite caused by the carpet viper *Echis ocellatus* are a major cause of envenomings. In northern Benin [21], Antivip-A was found effective at stopping bleeding within 2 hours in 60% of patients. Nine of 289 persons treated with Antivip-A died, including one girl who did not receive a full dose due to a shortage, six severe cases admitted with complications, and two cases believed to be bitten by a snake of the genus *Atractaspis spp*. In Paoua, Central Africa Republic [16], results with Antivip-A appeared less beneficial in a smaller cohort treated by MSF; five of 50 persons treated with Antivip-A died. Of note, in four of five cases, the second dose of two vials of Antivip-A was given with much delay, more than 12 hours after the initial dose. In 10 of 13 patients with visible bleeding, bleeding was not stopped within two hours following antivenom administration.

Antivip-A was also tested in Kindia, lower Guinea. All 118 patients treated with Antivip-A for what seems to be mild cytotoxic or mild haematotoxic envenomings survived [22]. However 4 of 22 patients treated for neurotoxic envenomation died. A susbsequent study in the same setting reviewed the efficacy of Antivip-A in an overlapping cohort of patients with neurotoxic syndrome caused by Elapidae [23]. The case-fatality rate in the groups treated with a low dose or high dose of Antivip-A was 15.4% and 17.6% respectively. An absence of clinical benefit was observed. Of note, assisted ventilation, a critical component of neurotoxic envenoming treatment, was not available.

Across these studies, a low rate of adverse events of between 10% and 15% was reported.

Summary: Conflicting results exist in relation to the effectiveness of Antivip-A for the neutralization of viperid bites in West Africa. In neurotoxic envenomings, Antivip-A showed poor results. Its safety profile appears good.

### Inoserp-P

One multicentre observational clinical study in northern Benin and in lower Guinea evaluated Inoserp-P in 209 patients [24]. A low case fatality rate, with one death among 109 patients was reported in lower Guinea, where neurotoxic envenomings represented 12% of admitted cases. In northern Benin, where many cases are caused by the carpet viper *Echis ocellatus*, four of 100 treated patients died. Blood coagulability was found to be restored within 24 hours in 98% of patients. Adverse events were reported in only 8% of patients.

Inoserp-P was also evaluated in Senegal in 63 patients [25]. It appeared to be effective and well tolerated, in spite of protocol deviations, including a lower initial dose than what is recommended. Blood coagulability was restored within 24 hours in 87.5% of patients. There were two deaths, including one neurotoxic pediatric case and one hematotoxic case in an adult presenting five days after the bite.

Summary: There is scant evidence available related to Inoserp-P. Inoserp-P seemed relatively effective in West African settings, and well tolerated.

### SAIMR-Poly

SAIMR-Poly is one of the most clinically-trusted antivenoms in sub-Saharan Africa, but there is paradoxically very little published material providing robust evidence of its efficacy. Most studies are of a poor quality, based on retrospective reports involving a small number of patients. Of the six studies included in this review [26, 27, 28, 29, 30, 31] SAIMR-Poly was used in 144 envenomed patients, and fatality was reported in only five cases.

More information is available on adverse events associated with SAIMR-Poly. An observational study in South Africa found that 13 of 17 patients who received SAIMR-Poly had severe early anaphylactoid reactions [27]. Varying rates of adverse events were reported in other publications of lower quality, with one publication expressing concerns over the high rates of adverse events [31].

Summary: There is limited evidence on the effectiveness of SAIMR-Poly. The rate of adverse events seems high.

### SAIMR-Echis

Three studies in the 1970s evaluated SAIMR-Echis in northern Nigeria [32, 33, 34], where *Echis ocellatus* is a frequent cause of envenoming. In a randomised controlled trial [34], the antivenom was found more effective in the treatment of carpet viper envenomings than a polyspecific antivenom then manufactured by Behringwerke. While no fatality was recorded in the 46 patients who received either antivenom, SAIMR-Echis reversed haematological abnormalities more rapidly and at a lower dosage than did Behringwerke’s antivenom.

Similar observations were made in two less robust studies in the same region [32, 33]. No fatality was recorded in the groups of 48 and 16 patients respectively who were treated for *Echis ocellatus* envenoming with SAIMR-Echis. In the latter group, bleeding was found to stop for the majority within 24 hours, although recurrent bleeding was observed in four patients a few days later.

Adverse side effects were reported in two of the three above studies. In one case series [33], 14 of 48 patients who received SAIMR-Echis had reactions. In the randomised controlled trial [34], immediate hypersensitivity was observed in four of 23 patients.

Summary: There is fairly robust evidence that SAIMR-Echis is able to treat the typical haemotoxic syndromes caused by *Echis ocellatus* envenoming. The rate of adverse events seems between moderate and high.

### VACSERA-Poly

One retrospective study reported mortality outcomes in 23 patients with prolonged clotting time in Gondar, north-west Ethiopia [35]. There were four deaths (17%) in this group. However, the number of vials that were given to the snakebite victims in this cohort was sub-optimal due to a short supply; most victims received between one and three vials, while between three and six vials were required according to clinicians.

Summary: There is one anecdotal report that suggests limited effectiveness of VACSERA-Poly in north-west Ethiopia.

### ASNA-D; Premium-A; Premium-CA; VINS-A; VINS-CA; SAIMR-Boom; VINS-Echis

We could not find any publicly available clinical evidence that met our inclusion criteria related to the remaining antivenom products listed in (Table 1).

## Discussion

There are relatively few clinical reports of antivenom use in sub-Saharan Africa in the scientific literature. While our strategy and inclusion criteria were broad in order to capture as many publications as possible, including studies with a weak methodology, only 26 publications were identified by our review. Many publications had to be excluded because the brand names of the antivenom products that were used were not reported, or because it was impossible to distinguish between patients treated with a specific antivenom product from patients treated with an alternative antivenom. In the future, authors of papers related to the treatment of snakebite should clearly mention the brand names of the products that were used, as well as doses and treatment times (if possible). If several antivenom products have been used during the course of a study, clinical data should be presented in a disaggregated manner, product by product.

The quality of the 26 studies that were included in our review was heterogeneous. Only two publications reported the results of a randomised clinical trial. Prospective observational cohort studies were more common. A substantial number of the publications included in this review were retrospective analyses of programmatic data. Unsurprisingly, study designs, clinical endpoints, and dosing strategies differed significantly between the studies.

Early and late reactions to antivenoms were monitored in only a few studies. The absence of reported side effects should therefore be viewed with caution, as it may simply indicate that side effects were not properly monitored.

In addition, the severity of envenoming upon admission and the time between bite and admission were very rarely reported, although both are known to have a very significant bearing on treatment outcomes.

It should be noted that the observed effectiveness of an antivenom in a specific geographical region should not be extrapolated to other regions where the cases of snakebite envenomation may be caused by snakes of other species. Most of the good quality prospective studies included in this review were conducted in West Africa, most often in the West African savannah where *Echis ocellatus* is recognised as the most medically important species. This is not a surprise as this is the sub-region in Africa with the highest burden of of snakebite envenomings [36]. However little is known about the effectiveness of antivenoms to neutralise envenoming by other species, not only envenoming caused by neurotoxic elapids, but also envenoming by other viperids, including other species of the *Echis* genus.

Similarly, the clinical ineffectiveness of an antivenom in a certain region should not be extrapolated to other regions. In particular, the clinical data reported on ASNA-C and VACSERA-Poly should be interpreted with caution: reports on ASNA-C came from the West African savannah, a region where *Echis ocellatus* is known to be the medically most important species, while ASNA-C was not developed against venom of *Echis ocellatus*. The report on VACSERA-Poly came from north-west Ethiopia, a region where *Echis pyramidum* and *Bitis arietans* are believed to be among the medically most important species, while VACSERA-Poly is not raised against *Echis pyramidum* or *Bitis arietans*. Acknowledging these limitations, our review was able to identify evidence related to three main groups of antivenom products.

Firstly, one polyspecific product (ET-Plus) and two monospecific products (ET-G, SAIMR-Echis) were tested in robust clinical studies and found to be effective against envenoming caused by the West African carpet viper (*Echis ocellatus*).

Secondly, four polyspecific products (Inoserp-P, FAV-A, SAIMR-Poly, Antivip-A) were evaluated only in observational single-arm studies, in some cases with mixed results.

Finally, five polyspecific products (ASNA-D, Premium-A, Premium-CA, VINS-A, VINS-CA) and two monospecific products (SAIMR-Boom, VINS-Echis) were not supported by any publicly available data that met our inclusion criteria. One polyspecific product (ASNA-C) was evaluated negatively in two clinical reports, one of which was of a good quality. Another polyspecific product (VACSERA-Poly) was also evaluated negatively in a retrospective case series.

### Conclusion

A minority of the antivenoms included in this review were supported by robust clinical data prior to their registration and commercialization in African countries. The absence of good quality, clinical effectiveness and safety data for the majority of these products is a major concern, as is the absence of publicly available pre-clinical data for some products. This inacceptable situation prompted the WHO to commission in December 2015 the preclinical testing of the different products intended for use in Africa in a systematic and blinded manner. The results of the assessment will be crucial to determine which products should be phased out, and which should be rolled out in the different sub-regions of Africa.

The overall dearth of clinical data on antivenoms intended for use in sub-Saharan Africa must be addressed. Comparative clinical trials should be implemented to compare the safety and effectiveness of products. Clinical trials must adopt a multi-centre methodology, in order to provide evidence of the effectiveness of products in different sub-regions and against different snake species. Many of the studies included in this review took place in rural hospitals and clinics with a long experience of snakebite management. With additional support, these sites could potentially host clinical trials.

For as long as anti-venom treatment is distributed in sub-Saharan Africa without adequate supporting clinical data, the safety and effectiveness of such treatment cannot be ascertained. Urgent investments in research are required to more accurately determine the regional specificity of existing forms of antivenom treatment. Additional financial and structural investments are required to ensure the sustained production and supply of antivenom as a priority intervention to reduce snakebite-associated morbidity and mortality across sub-Saharan Africa.
